# Spinal cord injury in abusive and accidental head injury in children, a neuropathological investigation

**DOI:** 10.1007/s00414-025-03418-0

**Published:** 2025-01-20

**Authors:** Michela Colombari, Claire Troakes, Andrea Verzeletti, Safa Al-Sarraj

**Affiliations:** 1 Institute of Legal Medicine of Brescia, University of Brescia, Piazzale Spedali Civili, 1, Brescia, 25123 Italy; 2 London Neurodegenerative Diseases Brain Bank, Institute of Psychiatry, Psychology & Neuroscience, King’s College London, London, UK; 3 Department of Clinical Neuropathology, King’s College Hospital NHS Foundation Trust, London, UK

**Keywords:** Abusive head trauma, Spinal cord injury, Spinal subdural haemorrhage, Spinal nerve roots, Forensic investigation

## Abstract

The diagnosis of abusive head trauma (AbHT) in children is a challenging one that needs to be differentiated from natural disease and accidental head injury (AcHT). There is increasing evidence from the Neuroradiology field showing spinal cord injury in children subject to AbHT, which has, so far, been poorly investigated pathologically. In this study we retrospectively reviewed the forensic records of 110 paediatric head injury cases over an eight-year-period. The records included detailed circumstances of death and clinical history alongside neuropathology, ophthalmic pathology and osteo-articular pathology. Based on the final multidisciplinary agreement, the 110 case were grouped into AbHT (*n* = 40), AcHT (*n* = 9), not clearly accidental or abusive (“undetermined” (UHT) *n* = 8) and non-traumatic brain injury (NTBI, *n* = 53). The spinal cord pathology present within each group was compared. Spinal subdural haematoma (SDH) was present in 71% of AbHT and 50% of AcHT cases and were located predominantly at the thoracolumbar level. In AbHT cases without spinal SDH, the suspected mechanism of injury was that of head impact rather than shaking, whilst cases of AcHT with spinal SDH were associated with direct trauma to the spinal cord. Injury of spinal nerve roots in AbHT was almost three times that seen in the accidental head injury group (58% vs. 17%). The study shows that pathological examination of the spinal cord and spinal nerve roots is of high value in investigating AHT and may help in differentiating AbHT from AcHT.

## Introduction

Abusive head trauma (AHT) in children is notoriously one of the most challenging diagnoses for the forensic pathologist. The American Academy of Paediatrics (AAP), in 2009, recommended using the term AHT over the formerly used designations (e.g. Shaken Baby Syndrome and Non-accidental Head Trauma NAHT) because of the different mechanisms of trauma. These include violent acceleration and deceleration of the brain resulting from forward and backward movement of the head and neck, as seen in Shaken Baby Syndrome, direct impact or a combination of these two mechanisms in what many termed shaken/impact syndrome [1]. The common morphological criteria for diagnosis of AHT in children include small amounts of multifocal subdural haematoma with particular predisposition for the interhemispheric fissure, cerebral oedema with hypoxic ischaemic changes and retinal haemorrhage. These criteria may be recognised as criteria which raises the suspicion of AHT. However, they have to be actively investigated and differentiated from natural disease which may cause similar features (such as haematological malignancy, septicaemia and prolonged hypoxic ischaemic brain injury causing disseminated intravascular coagulopathy), malformation such as fibrovascular dysplasia and from accidental head injury. For these reasons, the diagnosis of child abuse cases requires multi-disciplinary inputs from several clinical and pathological specialities including comprehensive clinical review, neuro-radiological examination, ophthalmic and osteoarticular pathology, haematological investigation and toxicology before a final conclusion can be made. There is now increasing evidence from the neuroradiology field that examination of the spinal cord can provide an important source of information regarding cause of injury and that spinal blood collection is highly suggestive of an abusive aetiology. The incidence of spinal blood collection in AHT is reported to be as much as 44–48% when all the spinal cord levels are analysed as opposed to just 0–18% when the assessment is performed at cervical level only; in correlation with evidence that the most frequent spinal SDH are located at the thoracolumbar rather than the cervical level [2–5]. Serinelli et al.., also observed that the amount of spinal cord injuries in AHT at the thoracolumbar level are almost double that at the cervical level [6]. In the clinical practice guidelines from the French Haute Autoritè de Santè, spinal cord lesions have been mentioned as a diagnostic criteria when seen together with intracranial subdural haematoma [7]. However, spinal cord injury in AHT children has been poorly investigated from a pathological point of view as the majority of diagnostic assessments are focused on brain and spinal cervical level only [8–14]. In this study we therefore carry out an assessment of pathology seen within the whole spinal cord in cases of AHT in children and investigate its value in differentiating abusive head tauma (AbHT) from accidental head trauma (AcHT).

## Materials and methods

We retrospectively reviewed the forensic neuropathological reports of all paediatric cases under the age of five years old from the registry of the Clinical Neuropathology Department at King’s College Hospital from January 2011 to June 2019. We collected an overall number of 113 cases; 50 females and 63 males (median age: 34,6 months). We reviewed the available description of the circumstances of the death, alongside the comprehensive neuropathological investigation of the brain and, when available, the spinal cord. All the cases of traumatic brain injury had been thoroughly investigated by a panel of experts in forensic, paediatric, osteoarticular and ophthalmic pathology in addition to a neuroradiology and clinical review by paediatricians and paediatric neurosurgeons. According to the final agreed diagnosis (as reached by a multidisciplinary panel of experts based on the clinical evidence rather than the legal/court verdict or conclusion of criminal or civil trials), the cases were divided into four categories: abusive head trauma (AbHT), accidental head trauma (AcHT), undetermined head trauma (where the given diagnosis and expert opinion was divergent or not certain) and cases of death not related to brain trauma (non traumatic brain injury category, NTBI). We reviewed the post mortem brain assessment of each case including the assessment of the dura, examination of the brain, the spinal cord (when available) and the histological and immunohistochemical stains. The latter include immunohistochemistry for β-APP (Beta Amyloid Precursor Protein) [MAB348, Millipore, 1:5000] and CD68 [M0876, Agilent Technologies, 1:50], both performed using the Bond automated staining machines. In 58 cases, the reports included a description of the spinal cord, from the cervical to varying lower levels. As with the brain, the description included the macroscopic examination of the spinal cord, before it was transversally sliced, together with the spinal meninges, to preserve any SDH and/or subarachnoid haemorrhages (SAH). Information on intracranial SDH, Retinal haemorrhage, Subdural haemorrhage, Subarachnoid Haemorrhage and βAPP immunopositivity was extracted for each case and analsysed between groups. For statistical analysis we performed the Fisher’s exact test.

## Results

**Fig. 1:**
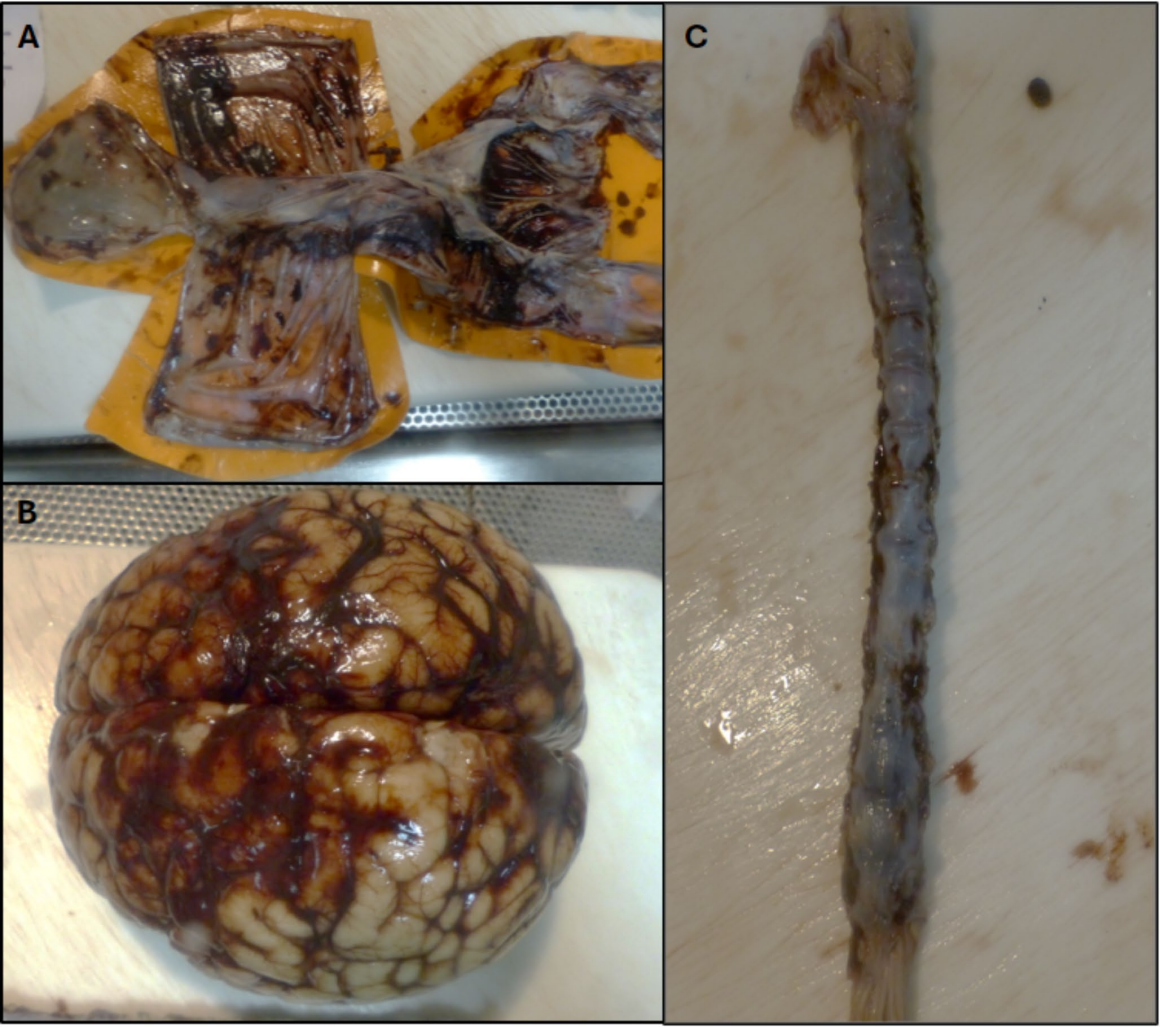
A. Dura (**A**), brain (**B**) and spinal cord (**C**) from a 5 month old female diagnosed with abusive head trauma. (**A**) multiple patches of thin SDH, underneath dorsal and infratentorial parts of dura; (**B**) moderately thick film of SAH covering the dorsal surface of the cerebral hemisphere in the parietal lobe and slightly in the occipital lobe and lateral surfaces around right and left Sylvian fissure extending to the anterior and inferior surfaces of temporal lobes; (**C**) dusky discolouration of outer surfaces of spinal cord consistent with SAH or SDH haemorrhage

**Fig. 2:**
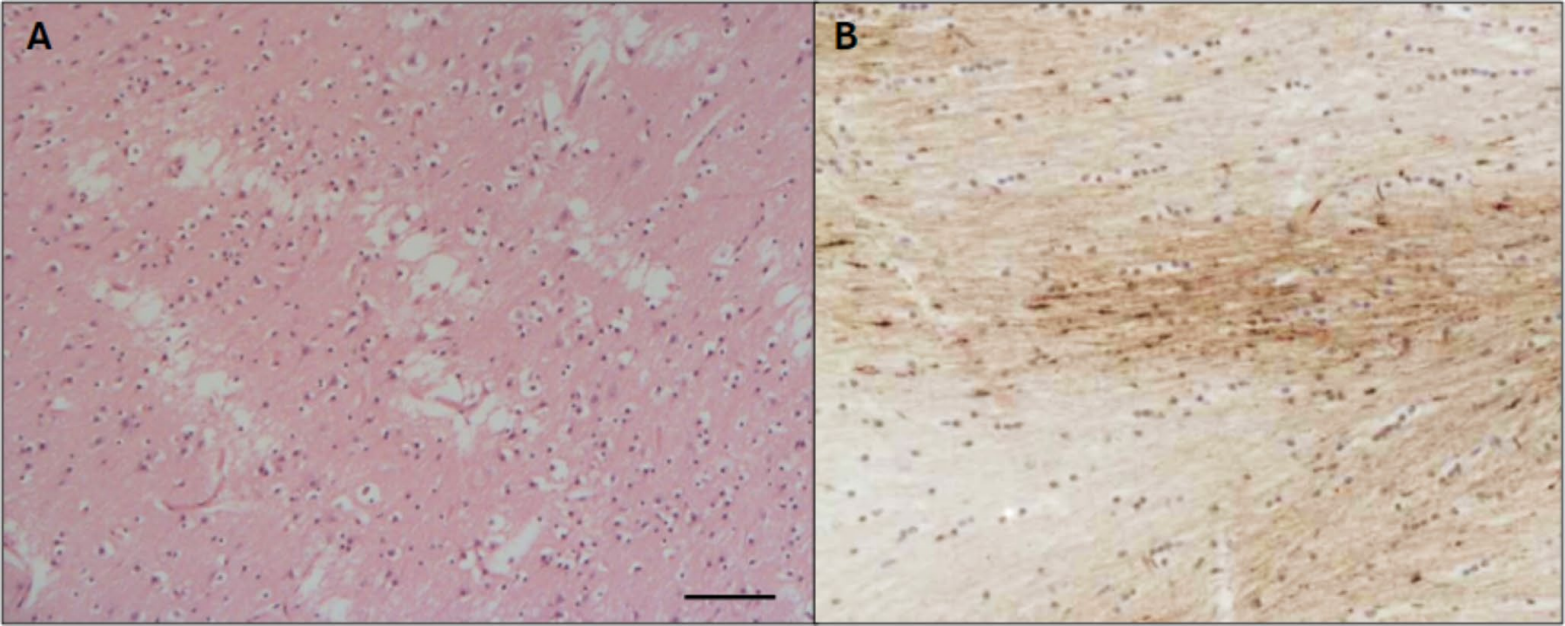
A: Haematoxylin and eosin-stained histological sections of cortex showing generalised recent ischaemic damage in the brain (vacuolation, shrunken neurones). B: βAPP deposition in the brain forming ill-defined areas with faint granular and filamentous deposits consistent with ischaemic disruption of axons. Scale bar represents 100 μm (**a**), 75 μm (**b**)

Three cases were removed from the initial group of 113 cases as information on circumstances of the death were not fully provided. From the remaining 110 cases (63 males and 47 females, mean age 36 weeks, ), 57 showed features of traumatic brain injury (51.8%), 40 of which were classified as AbHT and eight as AcHT group (Table 1). In the remaining nine cases, there was not a unanimous agreement between experts as to whether they were clearly accidental or abusive and these undetermined cases were thus not included in the final analysis. The control group consisted of 53 children who died from non-traumatic brain injury (NTBI) (e.g. hypoxia). Most of the 40 AbHT cases showed features of small amounts of multifocal intracranial SDH and of ischemic encephalopathy (Table 2) (Figs. 1 and 2). In one case it was not possible to ascertain the pathology of the dura due to unavailable histological sections (case n.38). The SDH appeared as a bilateral thinfilm of blood in the vast majority of cases (38/39), whereas in one case only a small amount of SDH was seen unilaterally distributed, on the right cranial dura (case n.20). According to the microscopic features, SDHs of different ages (old and recent) were diagnosed in 20/39 cases, recent SDH in 18/39 cases and old SDH in just one case. The brains were observed to be swollen and soft in consistency at the macroscopic examination and ischemia was microscopically confirmed in all cases except two (cases n.15 and n.16). Retinal haemorrhages and/or optic nerve haemorrhages were detected in 30 (97%) of the 31 cases with available information (Table 2); among them, 26 had retinal haemorrhages only, three showed blood around the optic nerve only and one case showed both retinal haemorrhages and blood around the optic nerves. The child without retinal haemorrhages was a 4.5-week-old male in whom the type of injury was judged by all experts to be an impact predominant type of injury without significant acceleration/deceleration of the head, thus giving a possible explanation for the absence of retinal haemorrhages (case n.17). In the AcHT group all the children presented with ischaemic features in the brain, while intracranial subdural haematoma was seen in 5/8 cases (Table 3). In these cases, subdural intracranial collections were explained on the basis of clear and witnessed circumstances which included a car accident, traumatic delivery and a dog biting the child on the head. The pattern of subdural collections in this group differ from the typical bilateral thin film and multiple locations seen in AbHT cases as they appear mainly localised and associated with contusions and lacerations in the cortex as well as epidural haemorrhages, intraventricular bleeding and skull fractures. The cases in this group had no information on retinal haemorrhages, except one (case n.2) which shows blood around the optic nerves at post mortem examination. In the 53 non-head trauma cases, ischemia was seen in eight cases and one case had subdural haematoma, which appeared as thin multiple patches of brownish discolouration. The circumstances of the death were not clear but jugded to be a possible previous incident of head injury or a consequence of birth related injury. The retinal haemorrhages were detected in 4 of the 12 cases with available information (33%) (Table 1). In those with retinal haemorrhages, three cases (males of 14 months, 2 months and 15 days of life) were reported to be found unresponsive and floppy and were taken to the hospital where they survived two, four and six days respectively. Brain examinations showed generalised hypoxic-ischaemia and brain swelling, in absence of signs of traumatic injury, therefore retinal haemorrhages were considered a direct result of brain swelling and a consequence of longstanding ischemic changes, or of disseminated intravascular coagulopathy. In one further case (male, 5 months old) the child was reported to have suffered from cardiac arrest 22 h before death. He presented unilateral retinal haemorrhage with no other evidence of traumatic injury. Overall, the combination of subdural haematoma, brain ischaemia and retinal haemorrhages were seen in 29/30 (97%) cases in the AbHT group as opposed to none of the children in the NTBI group.

**Table 1:** Number of cases, age and gender, Intraranial SDH, Brain edema/ischemia and retinal haemorrhages

	*N*.of cases	Median age (w)	Gender	Intracranial SDH	Brain edema/ ischemia	RH
AbHT	40	27,4 [1 d– 3 yr]	21 F/19 M	40/40 (100%)	40/40 (100%)	29/30* (97%)
AcHT	8	38 [1 d– 3 yr]	3 F/5 M	5/8 (62.5%)	8/8 (100%)	Not recorded for any case
NTBI	53	39 [1 d– 6 yr]	19 F/34 M	1/53 (2%)	4/53 (7%)	4/12* (25%)

*AbHT* Abusive Head Trauma, *AcHT* Accidental Head Trauma. *NTBI* Non Traumatic Brain Injury,*w* weeks, *d* days, *y* years, *F* female, *M* male, *SDH* Subdural Haemorrage, *RH* Retinal Haemorrhage, *cases with information on Retinal Haemorrhage

**Table 2:** Comprehensive table on AbHT cases, intracranial SDH, RH and spinal injuries

	Gender and age	Intracranial SDH	RH	Spinal SDH	Spinal SAH	Spinal nerve injuries
1	M 26 w	recent + old bilateral	bilateral	recent + old lower cervical, thoracic, lumbar, cauda equina	recent + old lower cervical, thoracic, lumbar, cauda equina	β-APP + cervical
2	F 9 w	recent + old bilateral	bilateral	recent + old Old: all levels Recent: thoracic and lumbar	recent thoracic and lumbar	Absent
3	M 2 w	recent bilateral	unilateral, seen as blood around the optic nerves	Absent	recent cervical, thoracic, lumbar	β-APP + cervical and thoracic
4	M 7 w	recent bilateral	Bilateral	recent cervical, thoracic and lumbar	Absent	β-APP + Level not mentioned
5	F 77w	old bilateral	bilateral, seen as blood around the optic nerves	old all levels	old all levels	Absent
6	F 9 w	recent bilateral	bilateral	recent all levels	recent lower cervical, thoracic, lumbar, cauda equina	Absent
7	M 17 w	recent + old bilateral	bilateral	recent thoracic and lumbar	recent thoracic and lumbar	β-APP + thoracic and cauda
8	M 99 w	recent + old bilateral	bilateral	recent all levels	Absent	β-APP + cervical, thoracic and cauda
9	F 30 w	recent + old bilateral	bilateral	recent T8 down to cauda equina, more intense in the upper and lower thoracic segments.	recent from C7 to cauda equina	β-APP + all levels
10	F 26 w	recent bilateral	bilateral	recent cervical, thoracic and cauda equina	recent cervical, thoracic and causa equina	β-APP + cervical, thoracic
11	F 104 w	recent bilateral	bilateral, seen as blood around the optic nerves	recent thoracic and lumbar.	recent thoracic and lumbar	β-APP + cervical
12	M 7 w	recent bilateral	bilateral	recent lower thoracic and lumbar	recent lower thoracic and lumbar	β-APP + thoracic
13	F 26 w	recent + old bilateral	not recorded	recent + old all levels	recent + old all levels	Absent
14	F 7 w	recent + old bilateral	not recorded	recent lower thoracic	Absent	Absent
15	F 0 w	recent bilateral	not recorded	Absent	Absent	Absent
16	F 16 w	recent + old bilateral	bilateral	Absent	Absent	β-APP Not assessed SAH
17	M 21 w	recent + old bilateral	absent	recent all levels	Absent	β-APP + thoracic, lumbar and cauda
18	M 47 w	recent + old bilateral	bilateral	recent Level not recorded	Absent	Absent
19	F 52 w	recent bilateral	bilateral	recent all levels	recent + old all levels	β-APP + all levels
20	M 156 w	recent unilateral	not recorded	not submitted SC	No SC submitted	No SC submitted
21	F 19 w	recent bilateral	bilateral	recent low cervical, thoracic and lumbar segment	recent all levels	Absent
22	M 13 w	recent + old bilateral	bilateral	recent all levels	recent all levels	Absent
23	M 6 w	recent + old bilateral	not recorded	recent + old all levels (recent at cervical and thoracic, old at thoracic and lumbar)	recent cervical	Absent
24	F 30 w	recent bilateral	bilateral, also seen around both optic nerves	recent all levels	recent all levels	β-APP + cervical, thoracic and lumbar
25	M 5 w	recent + old bilateral	not recorded	recent + old all levels	recent all levels	Absent
26	F 30 w	recent + old bilateral	not recorded	No SC submitted	No SC submitted	No SC submitted
27	F 17 w	recent + old bilateral	bilateral	recent + old all levels	recent not mentioned	β-APP + cervical
28	M 6 w	recent bilateral	bilateral	recent thoracic and lumbar	recent thoracic and lumbar	β-APP + thoracic
29	M 43 w	recent bilateral	bilateral	recent all levels, more obvious in the lumbar segment	recent all levels	Absent
30	M 11 w	recent bilateral	bilateral	recent lower thoracic	recent cervical, thoracic, lumbar	β-APP + cervical, thoracic and lumbar
31	F 4 w	recent + old bilateral	unilateral	recent + old all levels	recent + old all levels	β-APP + thoracic and lumbar
32	M 9 w	recent + old bilateral	not recorded	Absent	Absent	Absent
33	M 6 w	recent bilateral	unilateral	recent all levels	Absent (SDH only)	Absent
34	F 14 w	recent bilateral	bilateral	recent all levels	recent all levels	β-APP + cervical SAH - all levels
35	F 21 w	recent + old bilateral)	bilateral	recent lower thoracic and lumbar	recent lower thoracic and lumbar	β-APP + level not recorded
36	M 8 w	recent bilateral	bilateral	recent all levels	recent all levels	β-APP + all levels SAH lumbar
37	F 7 w	recent + old bilateral	not recorded	recent + old thoracic and lumbar	recent + old thoracic and lumbar	SAH thoracic and lumbar
38	M 60 w	undetermined bilateral	bilateral	Absent	Absent	Absent
39	F 30 w	recent bilateral	bilateral	recent all levels	recent all levels	Absent
40	F 21 w	recent + old bilateral	bilateral	recent + old all levels	recent all levels	β-APP + cervical, thoracic and lumbar
	**21 F** **19 M** **Median: 27.4 w**	**recent + old: 20** **recent: 18** **old: 1**	**Bilateral: 26** **Unilateral: 3** **not mentioned: 9**	**Cervical: 23** **Thoracic: 32** **Lumbar: 29** **Cauda equina: 22**	**Cervical: 20** **Thoracic: 26** **Lumbar: 26** **Cauda equina: 17**	**Cervical: 13** **Thoracic: 15** **Lumbar: 10** **Cauda equina: 7**

*AbHT* Abusive Head Trauma, *M* Male, *F* female, *w* weeks, *RH* Retinal Hemorrhage, *SDH* Subdural Hemorrhage, *SAH* Subarachnoid Hemorrhage, 0 absent, 1 present, *β-APP +* beta amyloid precursor protein positive immunohistochemistry, *N.* total number, *SC* Spinal Cord. All the cases were positive for intracranial ishemia

**Table 3:** Comprehensive table on AcHT cases, intracranial SDH, RH and spinal injuries

	Sex and age	Intracranial SDH	RH	Spinal SDH	Spinal SAH	Spinal Nerve injuries
1	M 1w	recent unilateral	not recorded	Absent	No SC submitted	No SC submitted
2	M 156 w	recent + old laterality not recorded	bilateral, seen as blood around the optic nerves	Absent	Absent	Absent
3	F 30 w	recent laterality not recorded	not recorded	recent all levels	recent all levels	Absent
4	F 86 w	Absent	not recorded	Absent	No SC submitted	No SC submitted
5	M 1 w	recent bilateral	not recorded	recent thoracic and lumbar	recent lumbar	Absent
6	F 39 w	recent unilateral	not recorded	recent thoracic	recent thoracic and lumbar	Absent
7	M 0 w	Absent	not recorded	Absent	Absent	SAH cervical and thoracic
8	M 1 w	recent + old bilateral	not recorded	1 recent thoracic	Absent	Absent
	**5 M/3 F** **Median**: **39 w**	**recent + old: 2** **recent: 2** **old: 0**	**Bilateral: 1** **Unilateral: 0** **not recorded: 7**	**Cervical: 1** **Thoracic: 4** **Lumbar: 2** **cauda equina:1**	**Cervical: 1** **Thoracic: 2** **Lumbar: 3** **cauda equina: 0**	**Cervical: 1** **Thoracic: 1** **Lumbar: 0** **Cauda equina: 0**

*AcHT* Accidental Head Trauma, *w* weeks, *SDH* Subdural Hemorrhage 0 absent, 1 present, *RH* Retinal Hemorrhage, *SDH* Subdural Hemorrhage, 0 absent, 1 present, *SAH* Subarachnoid Hemorrhage 0 absent 1 present, *N.* total number, *SC* Spinal Cord. All the cases were positive for intracranial ishemia

### Axonal injury in the brainstem

Pathology was investigated within the brain stem due to its linking role between the spinal cord and brain. Axonal injury was assessed via detection of axonal retraction balls (in H&E stained sections) and/or β-APP staining (Fig. 2). In the latter, β-APP deposition forming ill-defined areas with a filamentous and granular pattern of accumulation is consistent with ischaemic damage to the axons. While β-APP accumulation as well-defined globules (rounded and fusiform) and short filamentous structures individually scattered or in groups along white matter tracts is more consistent with traumatic damage [15]. The distinction between these patterns was not possible in some cases, therefore both ischaemic and traumatic damage to the axons are considered as possible causes for the axonal injury (here we use the term non-specific). In general, axonal injury was detectable through β-APP in the brainstem structures in the vast majority of AbHTcases (82.5%) and AcHT cases (87.5%) as opposed to just 9% of NTBI. The most common location of β-APP deposition was the pons-medulla area (85% AbHT, 100% AcHT, 80% NTBI) with a lower incidence of positivity in the midbrain (67% in AbHT, 0% from the AcHT and NTBI). In the AbHT group 33/40 were positive for β-APP in the brainstem, 14 judged to be ischaemic, 4 traumatic, 11 both traumatic and ischaemic and 4 non-specific. In the AcHT group 7/8 cases showed features of axonal injury of which 2 were ischaemic, 3 traumatic and 2 non-specific. Interestingly, retraction balls detected with H&E stain (indicating axonal injury) were seen in the cortico-spinal tract in the pyramids of medulla or in ponto-medullary junction only in the histological preparations from AbHT cases as opposed to none of the accidentally injured group.

## Spinal cord injuries

### Spinal blood subdural haemorrhages

**Fig. 3:**
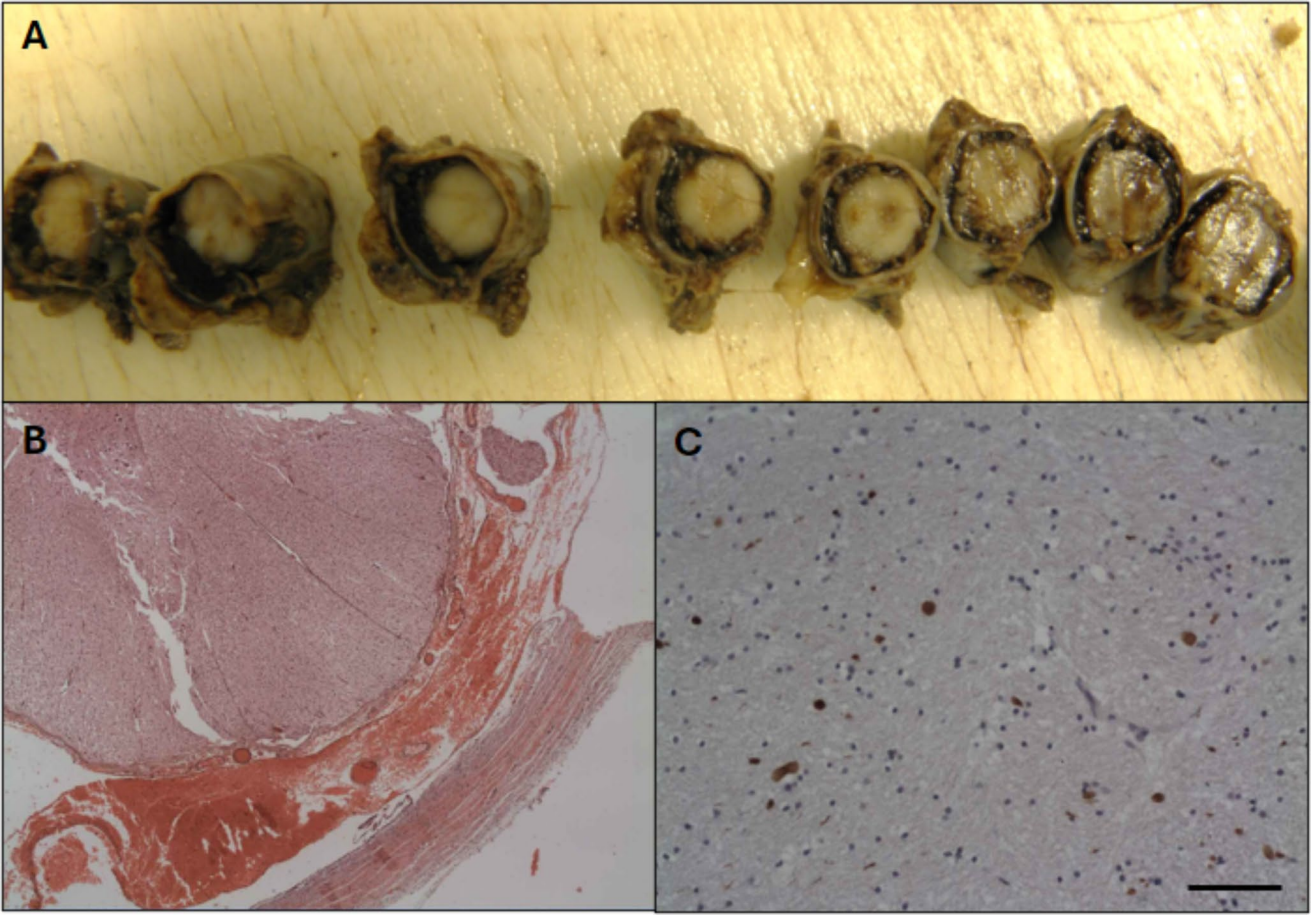
Macro (**A**) and micro (**B**,**C**) representation of spinal cord. **A**) material with reddish /dusky discolouration between the dura and the spinal cord consistent with SAH and SDH haemorrhages; **B**) histological examination shows recent haemorrhages in the subdural and subarachnoid spaces composed of well preserved red blood cells **C**) in the cervical segment, the βAPP staining shows multiple small rounded to oval-shaped accumulated deposit in the spinal cord’s white matter tracts extending to the subpial areas consistent with recent axonal injury. Scale bar represents 100 μm

Information about spinal cord examination was available in for 51 out of 57 of the head trauma cases and 17 out of 53 of the NTBI cases. Overall, blood collections (SDH and SAH) was seen in 71% from AbHT and 50% from AcHT. In the traumatic injury group spinal subdural haemorrhage (spinal SDH) was seen in 39 cases (76.5%) whereas in the NTBI cases only 2 of the 17 assessed spinal cords were positive for SDH (12%)– showing a significantly higher occurrence in the cases with traumatic head injury (AbHT, AcHT) than those without (NTBI) (*p* < 0,00001, Fisher’s exact test). The spinal SDH was more frequently seen in AbHT cases (33/38, 87%) than AcHT (4/6, 67%) (Table 4) (Fig. 3). The pathological assessment of the 5 AbHT children negative for spinal blood collections showed injuries caused by impact only mechanisms, with no pathological features of excessive hyper flexion /hyper extension of head and neck region. When the topographic location of SDH collections is taken into consideration, in both the AbHT and the AcHT the vast majority of cases showed SDH in spinal cord at the thoracolumbar level. In the AbHT 32 out of 37 cases with available information had SDH at thoracolumbar level versus 4 out of 6 AcHT and the lumbar location was seen in 29/37 AbHT and 3/6 AcHT. Spinal blood collections at cervical level had a small incidence in both AbHT and AcHT head trauma groups and was seen in 22/38 cases in the former and 2/6 in the latter. Interestingly, when subdural blood collections were detected at the cervical level it was consistently associated with blood collection at lower levels. The presence of subarachnoid blood collections (SAH) in the spinal cord appeared statistically related to traumatic aetiology (*p* = 0.0013) being seen in 31/44 traumatic brain injuries cases (70%) as opposed to just 4/17 NTBI (23.5%), however no significant differences in incidence was found between abusive and accidental head trauma. In the same manner as subdural blood collections, the presence of spinal SAH at a lumbar level was predominantly seen at thoracolumbar level (26/36 in AbHT and 3/6 in AcHT with available information).

**Table 4:** AbHT cases with no SDH and AcHT cases with SDH

Case number	Age and gender	Circumstances of the death	Extracranial findings	Intracranial findings	Spinal cord findings
AbHT cases with no SDH					
Case 3*	2 w, M	Alleged to have been found unresponsive	Skull fractures, blood around the optic nerves	Signs of **direct impact trauma** on the left side of the brain	EDH and SAH at all levels
Case 15*	1 d, F	Alleged to have been found dead	Skull fractures	SAH, signs of **direct impact** trauma to the left frontal and left parietal lobes	Spinal cord examination was negative except for SAH in the spinal nerve roots at the cauda equina section
Case 16*	4 m, F	Alleged to have been found dead	Multiple bruises on scalp, ears, thighs and knees	SDH, SAH, brain swelling, signs of **direct impact**	Scattered patches of EDH, SAH
Case 32*	2 m, M	Alleged to have been found unresponsive	Bruises all over the body	SDH, SAH, brain swelling, signs of **direct impact**	Spinal cord examination was negative except for EDH, SAH and SDH
Case 38*	14 m, M	Alleged to have been found unconscious and unresponsive	Bruises on face, head, skeletal fractures and retinal haemorrhages	SDH, SAH, signs of severe **direct impact** at the back of the head.	Patchy extradural haematoma in absence of SDH
AcHT cases with SDH					
Case 3^#^	F, 7 m	Car accident	Not mentioned	Tiny fragments of SAH in patches, intraventricular bleeding	EDH, SDH, SAH at all levels
Case 5^#^	F, 7 d	Complicated delivery	Skull fracture, multiple bruises on face	EDH, SDH, SAH	SDH, SAH in the upper and mild thoracic and lumbar segments
Case 6^#^	F, 9 m	Car accident	Not mentioned	SDH, SAH, contusions	Small amount of SDH and SAH in the thoracic segments
Case 8^#^	M, 8 d	Complicated delivery	Skull fracture, multiple bruises on scalp	SDH, SAH, multiple areas of hemorrhage	Small amount of SDH and SAH in the thoracic segments

*AbHT* Abusive Head Trauma, *AcHT* Accidental Head Trauma, *w* weeks, *d* days, *m* months, *F* female, *M* male, *SDH* Subdural Haemorrhage, *SAH* Subarachnoid Haemorrhage, *EDH* Extradural Haemorrhage. *Case number according to Table 2, ^#^Case number according to Table 3

### Spinal cord parenchyma injuries

Four out of 38 cases of AbHT showed softening and greyish discolouration of the central grey matter, while haemorrhage was seen in only one case at C2-C3 level. However, loss of neurons, shrunken neurons and an increased number of microglia cells were consistently seen on histological sections, supporting the common finding of ischemia features both in AbHT (31/38) and in AcHT (3/6), with no significant difference between these two groups. Spinal cord parenchyma involvement was investigated using β-APP immunohistochemistry which showed accumulated deposits in up to 68% (29/38) of spinal cords in AbHT cases, mainly as ill-defined areas of a granular and filamentous like pattern of accumulated deposits consistent with ischaemic disruption of axons. Ischemic patterns of β-APP had similar characteristics in the AcHT group.

### Spinal nerves injuries (β-APP deposition, SAH)

Investigation of the spinal cord histological features showed frequent involvement of nerve roots in association with trauma, such as β-APP accumulation and subarachnoid haemorrhages. The combined signs of spinal nerve involvement (β-APP deposition and subarachnoid haemorrhages) were found to be similarly related to head trauma (*p* = 0.001), being present in 49% of the traumatic (AbHT and AcHT) and just 6% of the non traumatic cases (NTBI). Nevertheless, the incidence in AbHT group was higher than in the AcHT group (58% vs. 17%). In the 19 AbHT cases with spinal nerve involvement and level information, the vast majority of them were at cervical (68%) and thoracic (79%) rather than lumbar (53%) and at cauda levels (38%). The majority of spinal nerve axonal injury lesions in the AbHT group were detected with β-APP immuno-reactivity (20/22), while few cases showed SAH (5/22). One case only in the AcHT group showed β-APP in spinal nerves, at cervical and thoracic level, while none showed any SAH around spinal nerve roots.

### Spinal soft tissue injuries and ligaments

Considering the available information regarding spinal soft tissue injuries and ligaments, 8/17 (47%) AbHT showed haemorrhages in paraspinal and sternocleidomastoid muscles. In the AcHT and NTBI groups information on muscle-skeletal injures was poor or not available. In the former, one case only showed haemorrhages in the soft tissue of the neck extending from the scalp. In the latter group, in the nine cases with spinal muscle-skeletal information, four had haemorrhage on the anterior neck and sternotyroid muscles.

## Discussion

Abusive head trauma is often a challenging diagnosis in the forensic setting. The features of brain ischaemia, intracranial subdural blood collections and retinal haemorrhages require further corroboration and review from multidisciplinary teams of forensic pathologists, paediatric pathologists, paediatricians, neuro radiologists, osteoarticular pathologists and many other experts before a clinical verdict is given to assist the legal investigation and courts [7, 16, 17]. The present study aimed to examine spinal cord changes from the neuropathological point of view, which is not adequately investigated in some cases, in order to determine additional pathological changes that would support the diagnosis and that may help to differentiate a case of abusive head trauma from a natural diseases and accidental injuries. The evidence from our study showed thatblood collections of both SDH and SAH are more frequently seen in AbHT (71%) cases compared to AcHT (50%) seen in AbHT and AcHT and (specifically 87% vs. 67% for SDH and 75% vs. 50% for SAH). The results are in agreement with the evidence from the neuroradiology field showing that SDH in spinal cord is usually observed in cases of abuse [2, 18–24] and with French Haute Autoritè de Santè (2017) including the spinal cord lesions as a diagnostic criteria for abusive head trauma cases [7]. There are cases of AbHT without spinal cord SDH in our cohort, but looking carefully into the five cases where no SDH is seen (Table 4), one can determine good evidence of head injury with predominant impact mechanism and less likely shaking (as per unanimous agreement of multi-disciplinary experts). These cases include those with clear head impact with skull fracture, contusions and laceration in the brain. Equally, the four cases of AcHT with SDH have clear and witnessed explanation of direct trauma to the spinal cord such as car accident, difficult delivery and dog biting (Table 4). In our experience (but not presented in the current cohort), spinal SDH may also be present in cases of AcHT if it is associated with a large cranial space occupying SDH that could potentially force itself to track down to reach the spinal cord dura. However, in these cases the SDHs are commonly large and unilateral, therefore having a different pattern from the pathology of thin and multi-located cranial SDH seen in AbHT, which are small in volume and unlikely to have caused enough pressure to tract down through many anatomical obstructions in the dura connecting the brain and spinal cord. Our results support the view that if there is no large cranial SDH or clear explanation of direct trauma on the spinal cord, then the spinal SDH is highly suggestive of inflicted rather than accidental head trauma. We found that spinal blood collections were mainly seen at the thoracolumbar level, in agreement with the increasing evidence from neuroradiology [2, 18, 24] and with previous neuropathological studies [6, 25].

### The origin of the spinal SDH

**Fig. 4:**
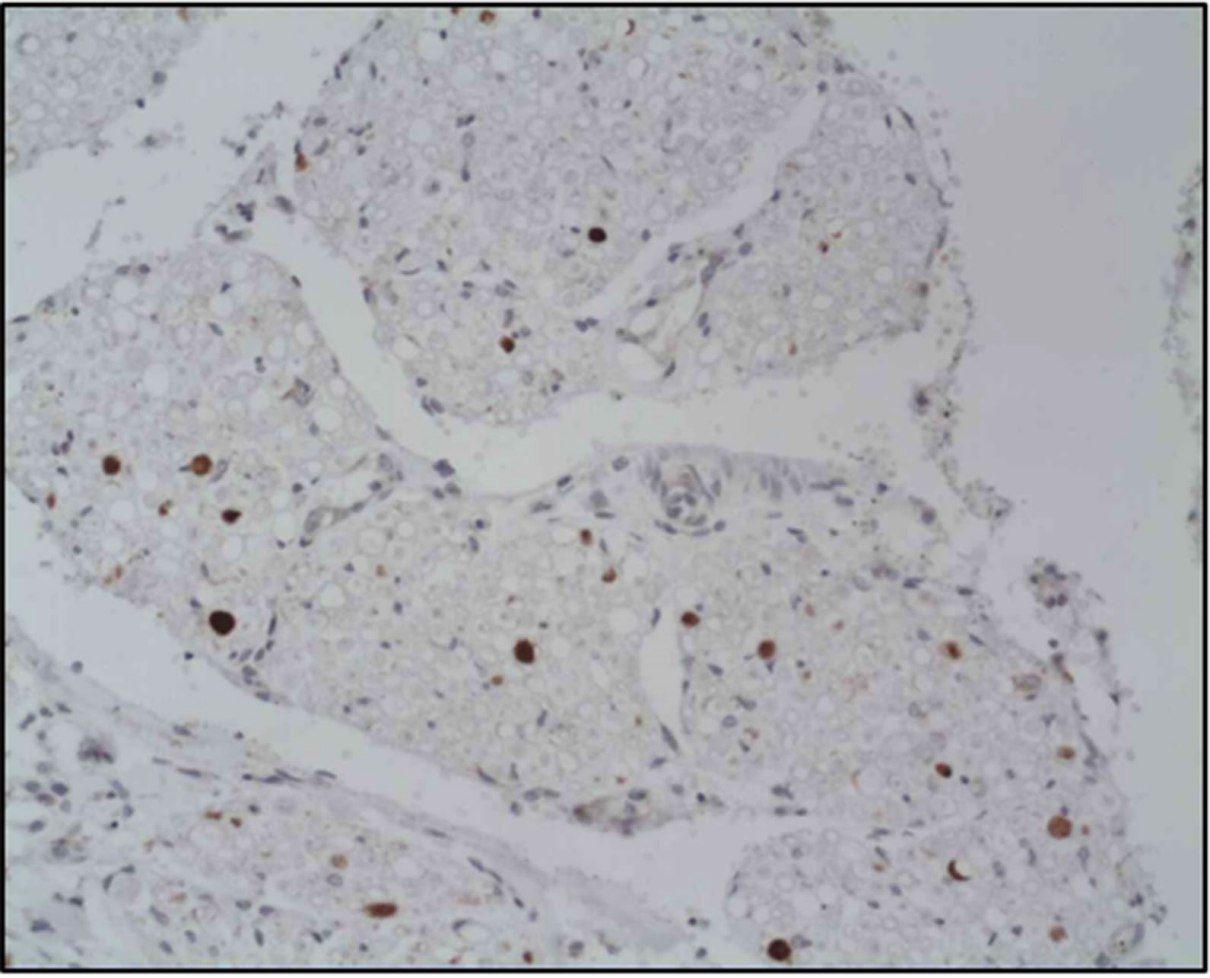
βAPP deposition indicating axonal injury in the spinal nerve roots. Scale bar represents 75 μm

In order to find additional evidence supporting the diagnosis of AHT, a complete investigation of the spinal cord should also include studying the spinal nerve roots. According to the results from our study the incidence of combined changes at the level of spinal nerve structures (β-APP accumulations and subarachnoid haemorrhages) in the AbHT group is almost three times that seen in the AcHT group (58% vs. 17%) (Fig. 4). The presence of injuries in spinal nerves supports the hypothesis that the traumatic damage to the spinal radicular arteries, which travel close to the spinal nerve roots, is a potential source of blood collection in the spinal cord, as proposed by others [26, 27]. It is possible that the excessive/violent forward and backward movement of the head and neck (such as in shaking) may lead to violent movement of the spinal cord in upward and downward directions within the spinal canal causing strain of the spinal nerve roots and the radicular arteries resulting in SAH, SDH and traumatic spinal nerve roots injury. The main parenchymal spinal cord change seen, namely the ischaemic damage, may be secondary to generalised central nervous system ischaemia but it may also be part of more localised damage caused by traumatic strain on the vascular supply, similar to that causing the SAH, SDH and the spinal nerve roots injury. Significantly, in our study the cervical location of blood collections was consistently seen in association with blood collections at lower levels, supporting the hypothesis of a primary blood source in the upper spinal levels. Although the results showed the lumbar location of blood collection in the spinal cord is statistically associated with abuse, the origin of the bleeding may not be restricted to one location and the bleeding may track down the spinal canal to include other lower locations.

### Brainstem and the mechanism of damage

Although the brainstem and spinal cord parenchymatous injuries may be of little help in distinguishing the aetiology of traumatic injury, as both the AbHT and AcHT presented similar features of ischemia, these could possibly be of some utility. The ischemia damage is a well-known feature in AbHT cases when examining the brain parenchyma [28]. Interestingly, in our study, retraction balls in the brainstem, which are a well-known sign of traumatic damage to the axon, were seen only in AbHT cases as opposed to none of the accidental cases, and they were located in the pyramids of medulla or ponto-medullary junction. These results are similar to previously published work by Geddes et al. [13] and Matschke et al. [29]. The systemic review from the United Kingdom’s Royal College of Paediatrics and Child Health (RVPCH) found that traumatic axonal injury is specific for AbHT (OR 2.2, 95% CI 1.2–3.9) and that the axonal damage has an active role in the onset of hypoxic-ischaemic changes intracranially observed in the AbHT cases [16]. Therefore our findings support the previously proposed hypothesis of primary injury of inflicted head trauma of shaking (or similar violent hyperextension/flexion of the cervical area) involves the cranio-cervical junction that could possibly cause traumatic strain and dysfunction of the cardio respiratory centre (located in the reticular formation of medulla) leading to apnoea and cardiac arrest, followed by the hypoxic/ischaemic damage. One of the limitations in the study was the lack of data on spinal soft tissue and ligaments and vertebra injuries in most cases. The later type of investigation was not routinely done before 2018 which the period we collect the cases for this study but it would certainly add further evidence in supporting the diagnosis of AbHT. Evidence from Neuroradiology supported that the craniocervical junction plays a fundamental role in the mechanism of intracerebral damage, as Choudhary et al. in 2014 saw that AbHT had evidently higher cervical ligamentous injuries (78% vs. 46% of the AcHT) and that there was a significant association between cervical ligamentous injury and the presence of cerebral ischaemic injury [19]In conclusion, our data adds evidence in confirmation of the recent consensus statements of several professional medical societies that the pathological examination of the whole length of the spinal cord appears of great importance - in combination with other pathological findings - to support the diagnosis of AHT.

### Conclusion and proposed pathological protocol for spinal cord examination in suspected child abuse

In conclusion, our data adds evidence in confirmation of the recent consensus statements of several professional medical societies that the pathological examination of the whole length of the spinal cord appears of great importance - in combination with other pathological findings - to support the diagnosis of Abusive Head Trauma. When spinal blood SDH is seen in a case of child head trauma, it’s important to search for history of direct trauma on the spinal area and if the data is negative, the possibility of abuse should be taken into consideration. Furthermore, when abusive head trauma is suspected, the absence of SDH in spinal cord does not exclude the abuse diagnosis and a direct impact trauma should be investigated. Thus, whenever a forensic pathologist is involved in the paediatric post-mortem examination suggestive for AbHT, especially when the child is under two years of age, the whole length spinal cord should be removed and analysed together with the spinal dura mater [17, 30]. The formalin fixed spinal cord should be transversally cut together with the dura according to the site of each of the segments, to preserve the anatomy of the structure of spinal nerve roots and any small amounts of SDH which might be lost if the dura is cut longitudinally. We suggest that at least 3–4 histological samples should be taken from cervical, upper and lower thoracic segments and two from lumbar and cauda equina. β-APP immunohistochemistry should be routinely performed together with CD68 (for macrophages and microglial cells) and Perl’s (for hemosiderin) to assess the spinal nerve roots and spinal cord parenchyma. To complete the examination, the cervical vertebra with the surrounding paravertebral muscles and tissue is also important to be removed and examined thoroughly after decalcification to assess any pathology such as fractures or muscle and ligamentous injuries. Finally, the Neuropathological changes from the spinal cord examination should be interpreted together with information on the circumstances of the death, especially in cases with a history of a high energy trauma to the head and spinal cord. A unified and validated procedure between different worldwide laboratories appears a fundamental stage in the progression of a consensus for diagnosis of AbHT in children.
